# Dynamic Behaviour of Donor Specific Antibodies in the Early Period Following HLA Incompatible Kidney Transplantation

**DOI:** 10.3389/ti.2022.10128

**Published:** 2022-04-11

**Authors:** Mason Phillpott, Sunil Daga, Rob Higgins, David Lowe, Nithya Krishnan, Daniel Zehnder, David Briggs, Natalia Khovanova

**Affiliations:** ^1^ School of Engineering, University of Warwick, Coventry, United Kingdom; ^2^ St James’s University Hospital, LTHT NHS Trust, Leeds, United Kingdom; ^3^ Warwick Medical School, University of Warwick, Coventry, United Kingdom; ^4^ NIHR Leeds In-Vitro Diagnostics Co-operative, Leeds, United Kingdom; ^5^ Histocompatibility and Immunogenetics, NHS Blood and Transplant, Birmingham, United Kingdom; ^6^ University Hospitals Coventry & Warwickshire NHS Trust, Coventry, United Kingdom; ^7^ North Cumbria Integrated Care NHS Trust, Carlisle, Cumbria, United Kingdom; ^8^ Institute of Cancer and Genomic Sciences, University of Birmingham, Birmingham, United Kingdom

**Keywords:** kidney transplantation, antibody dynamics, 5 years graft failure, donor specific antibody, dynamic patterns, clustering

## Abstract

In HLA-incompatible kidney transplantation, monitoring donor-specific antibodies (DSA) plays a crucial role in providing appropriate treatment and increases kidney survival times. This work aimed to determine if early post-transplant DSA dynamics inform graft outcome over and above other predictive factors. Eighty-eight cases were classified by unsupervised machine learning into five distinct DSA response groups: no response, fast modulation, slow modulation, rise to sustained and sustained. Fast modulation dynamics gave an 80% rate for early acute rejection, whereas the sustained group was associated with the lowest rejection rates (19%). In complete contrast, the five-year graft failure was lowest in the modulation groups (4–7%) and highest in the sustained groups (25–31%). Multivariable analysis showed that a higher pre-treatment DSA level, male gender and absence of early acute rejection were strongly associated with a sustained DSA response. The modulation group had excellent five-year outcomes despite higher rates of early rejection episodes. This work further develops an understanding of post-transplant DSA dynamics and their influence on graft survival following HLA-incompatible kidney transplantation.

## Graphical Abstract

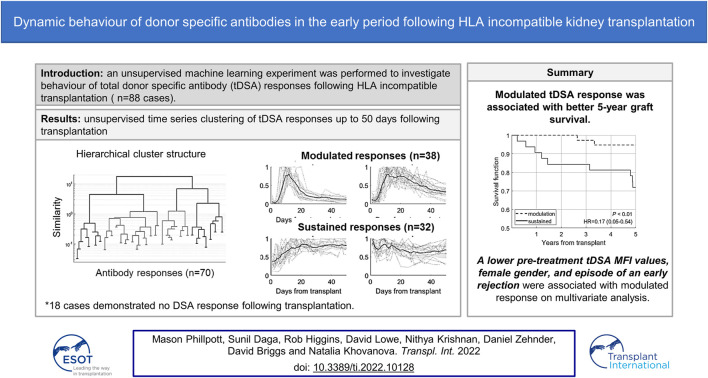

## Introduction

Despite the advances in the identification of acceptable mismatch programmes (1), better allocation of deceased donor kidneys (2), and advances in kidney sharing protocols for those with living kidney donors (3), there is still a role for HLA-incompatible transplants, especially when lower-risk scenarios could be identified (4) and those at the highest end of the sensitisation spectrum still do not have equal access to transplantation (5).

It is well-recognised that the presence of donor HLA specific antibodies (DSA) both before and after kidney transplantation correlates strongly with poorer graft outcomes (6–9). However, the monitoring and characterisation of the early post-transplant DSA response and how this may inform outcome and transplant management is still a developing area (10). Previous research has shown that DSA measurements pre-transplantation or at the time of transplantation can be a powerful tool for predicting graft outcome, but the sensitivity and specificity obtained varied with different DSA cut-off values (11–15).

Post-transplantation tools, such as protocol biopsies, in the early period to guide management and predict outcomes are limited and often not acceptable to patients. Early episodes of antibody mediated rejection (AMR) may be associated with recurrent rejection, chronic AMR and poor graft survival (16). The presence of DSA post-transplantation was associated with an increased likelihood of AMR (17–19) and graft failure (12, 20, 21). Recent studies (22, 23) suggest AMR may not always be associated with poor middle- or long-term graft failure (GF).

Studies on monitoring DSA immediately following transplantation are usually limited in the number of post-transplant samples (10, 17, 18, 24). The early post-transplant period (first 2 weeks) is a critical time for B-cell anamnestic memory and dynamic DSA behaviour and the occurrence of accelerated AMR episodes. The behaviour of DSA in the first month after transplantation and their associations with immediate/short term transplant outcomes has been previously described (25, 26). With access to up to 50 days post-transplant DSA measurements, our work looks at the medium-term outcomes and aims to determine how different dynamic DSA patterns relate to 5-year graft survival.

## Patients and Methods

### Patients

133 patients referred from multiple centres in the United Kingdom and Republic of Ireland for HLA-incompatible kidney transplantation between 2003 and 2014 at the University Hospitals of Coventry and Warwickshire NHS Trust were considered. Of these cases, 88 were used in the final analysis. Twenty-four cases were excluded for the following reasons: no consent to use of data (*n* = 1), not proceeding to transplantation (*n* = 7), also ABO-incompatible (*n* = 16), early death or early graft failure (*n* = 5), insufficient follow up data (*n* = 9), antibody assay saturated (*n* = 2), less than 5 years follow-up (*n* = 5). Within 5 years following transplantation, graft failure occurred in 13 out of the 88 cases, and all failed due to immunological reasons. Study approval was obtained from the local ethics committee (CREC-055/01/03 and 13/WM/0090).

### HLA Testing

HLA Class I and Class II specific antibodies were identified before transplantation by bead assay (One Lambda Inc. Canoga Park, CA), initially using HLA phenotype beads (N = 19) and subsequently with single antigen beads (SAB) as previously described for this programme (20). HLA typing of patients and donors was performed by a DNA probe assay (Lifecodes HLA SSO, Immucor) at a resolution comparable to the antibody identification, allowing identification of all donor-specific antibodies corresponding to HLA-A, -B, -Cw, -DRB1/3/4/5, -DQ, and -DP.

### Desensitisation and Immunosuppressive Protocol

68/88 patients required several sessions of pretransplant double filtration plasmapheresis (DFPP) with a target of negative FC cross-match or cumulative DSA median fluorescence intensity (MFI) <3,000 pre-transplantation. The maximum number of sessions administered was seven, with patients typically receiving five. In some cases where the DFPP sessions could not achieve a negative FC cross-match, patients were transplanted in the presence of higher DSA levels. IVIg was given in three cases. Twenty cases proceeded to transplant without DFPP because the total DSA MFI values were below 3,000 (*n* = 14) or with higher levels in cases of deceased donors’ kidney transplantation where pretransplant antibody reduction was not logistically possible (*n* = 7). For these deceased donor cases, DSA were predominantly specific from HLA-DP mismatches.

Typical immunosuppression consisted of 1,000 mg mycophenolate mofetil twice daily, starting 10 days before transplant with dosage reduced if white cell count dropped below 4.0 x 10^9^ per litre. Daily administrations of tacrolimus were commenced 4 days before transplantation. Dosages were given at 0.15 mg/kg/day in increments with a target trough level of 10–15 μg/L in the first month. At the point of surgery, a single 500 mg methylprednisolone dose was provided intravenously, and 20 mg basiliximab induction was given twice on day zero and day four post-transplant. Oral prednisolone was given at 20 mg/day and tapered to 5 mg/day after 30 days.

### Monitoring and Management Following HLA-Incompatible Kidney Transplantation

Apart from on-table post perfusion samples, a biopsy was done for cause only, i.e., in cases of graft function deterioration or creatinine stuck as described previously (27). Acute rejection (*n* = 41) episodes occurring before 30 days post-transplant were identified at incidence under the most recent BANFF guidelines (28). In six instances where the biopsy was not possible, for example, for patients on anticoagulation therapy or during weekends, a rapid rise of HLA DSA MFI values alongside a drop in urine output and increase in creatinine (with one case with delayed graft function on dialysis) was defined as clinical rejection. All 41 rejection cases were treated with a course of pulse methylprednisolone 500 mg once a day for 3 days. In thirty cases, a lymphocyte-depleting agent (ATG, OKT3, or Campath) was administrated. DFPP treatment was performed in thirteen cases, of which five were given IVIg. One case had ecluzimab in addition to rescue therapy (see Supplementary Table S1). DFPP was only given in cases with rejection and not pre-emptively, with DSA levels going up in the presence of good urine output and stable renal function.

### Pre-Processing of HLA DSA Data

The total number of DSAs in our cohort of 88 cases was 211, with between 1 to 7 for each patient. In this work, the levels in each case were considered. For the following analyses, we calculated the sum of individual HLA DSA MFIs to give a total DSA (tDSA) for each time point.

**FIGURE 1:**
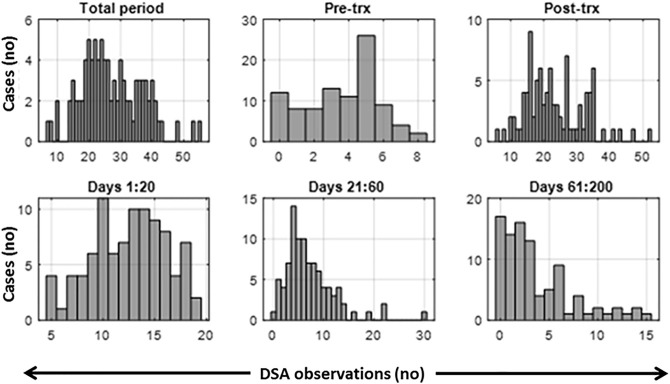
Histograms of the observed number of data points per measurement period. Total period includes pre-transplant and post-transplant period. Trx, transplant.

Post-transplant antibody testing involved more frequent testing during the early phase, with the majority (71/88) being sampled ten or more times during the first 20 days, after which the rate of sampling declined (Figure 1). Cases that had at least 21 days of DSA monitoring data points were included in this study. Variation in sampling days presents a challenge to clustering algorithms requiring uniform sampling rates. A linear interpolation was used within the 50-day time frame to fill missing values which were few in the first 2 weeks post-transplantation and increased with time (Figure 1). At the 2 week mark, the median length of interpolated values is 0 days; at 4 weeks, this extends to 2 days, at 50 days to 5 days, and at 100 days to 16 days. We, therefore, included up to 50 post-transplant days in the study; this extends well-passed the period involving the DSA rebound and avoids the times with high levels of missing data.

### Clustering of DSA Time Series

**FIGURE 2:**
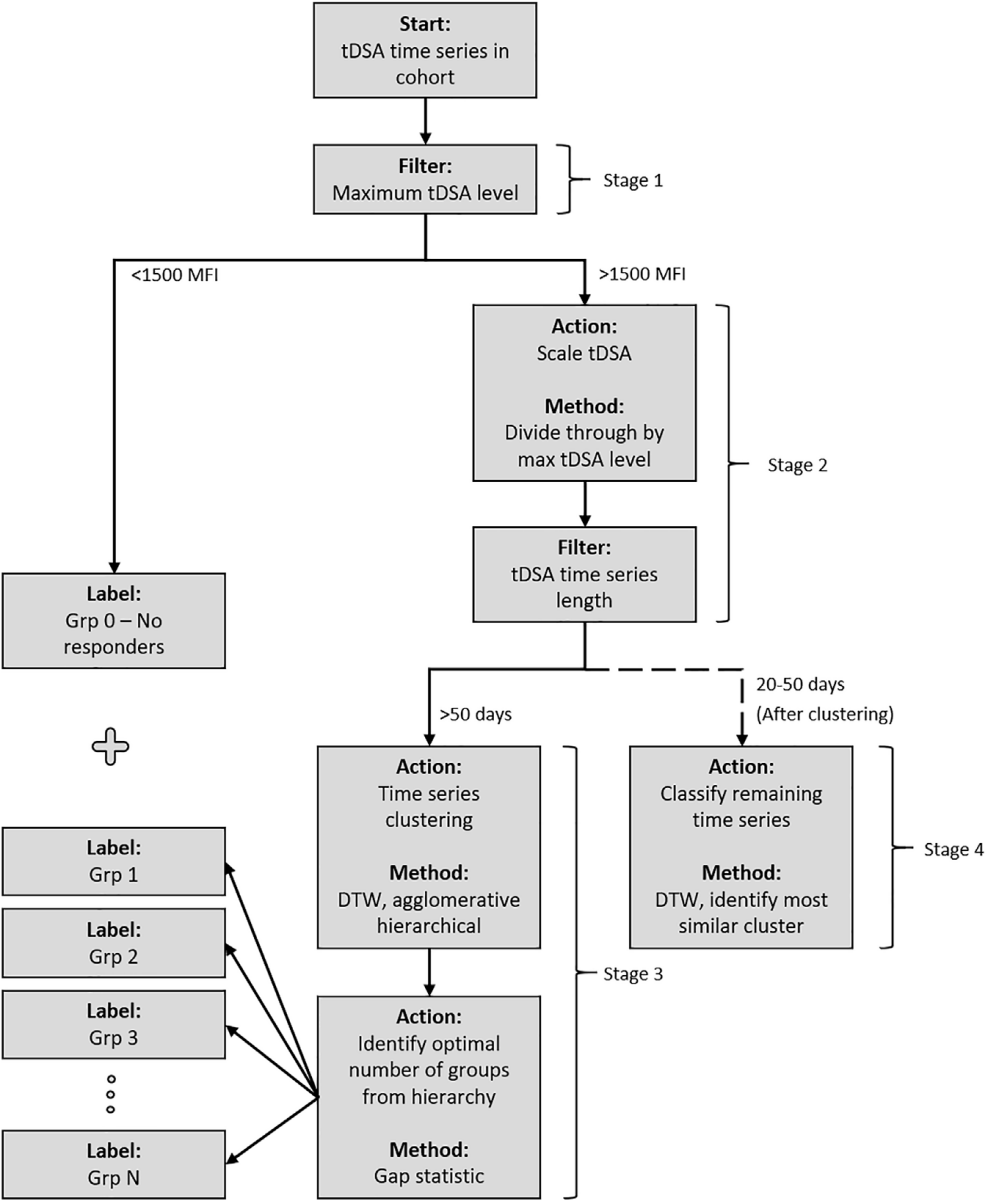
Post-transplant DSA dynamics clustering methodology. The cohort is passed through the system and sorted into groups based on the similarity of post-transplant DSA dynamics. Stage 1 separates DSA into a pre-designated “no response” group if their maximum post-transplant tDSA titre level is less than 1500 MFI. Stage 2 scales the remaining DSA based on maximum post-transplant tDSA titre level before an additional filter is applied, separating the DSA based on time series length. All data which have 50 days are passed to Stage 3 and subsequently clustered using DTW distance measure under an agglomerative hierarchical structure. The optimal numbers of groups within the structure are identified using the gap statistic. Stage 4 classifies the remaining DSA, which have 20–50 days of data, into the groups identified in Stage 3.

The DSA data are a time series comprising successive DSA measurements for 88 patients for up to 50 days post-transplant. These were investigated to identify possible classes of early post-transplant antibody behaviour (such as rebound and modulation). They could then be tested for association with pretransplant parameters and post-transplant events. The classification was performed using unsupervised machine learning clustering (29). The four-stage procedure, illustrated in Figure 2, was as follows.

Stage 1 Grouping of no-response data. No-response cases consistently demonstrated low tDSA, i.e., below 1500 MFI (N = 18). These cases are not used in the clustering algorithm due to the disruptive influence on the analysis once scaled. The DSA levels in this group also have a significant uncertainty (29, 30, 31), which leaves dynamics in this MFI range unidentifiable. Instead, they are considered as a separate no-response group 0.

Stage 2 Pre-processing for DSA time series clustering. Some post-transplant DSAs displayed little dynamic activity but high MFI levels. To distinguish such DSA dynamic patterns from one another without affecting the clustering algorithm, the time series were scaled by their corresponding maximal DSA levels (29). Additionally, many temporal clustering techniques require equal length time series, which in this case required establishing a defined period for clustering analysis. Picking a shorter time series length for analysis would allow for the inclusion of more cases in analysis, although at the compromise of losing potentially valuable data obtained from clustering longer time intervals. DSA time series length is inconsistent in our cohort, and a steady drop in measurements/samples is observed as time progresses. A period of 50 days provided the best compromise for analysis. The remaining cases were split based on their length: up to 50-days data available (longer time series) and 20–50 days (shorter time series).

Stage 3 DSA clustering and validation of the longer DSA time series (*n* = 47). Unsupervised clustering was completed via a dynamic time warping (DTW) distance measure (29) and agglomerative hierarchical approach (32). Cluster links were formed via the mean of the two joining time series. The algorithm identified the two most similar time series at each iteration based upon the distance measure and merged to form a cluster. The identified time series were then replaced by the newly formed cluster for which distance measures were newly calculated. For the unsupervised clustering, the number of groups was not known *a priori*. Although this value can be identified through visual inspection in some cases, additional certainty in estimation was required due to time series complexity. Here the gap statistic was implemented, which is a measure that estimates the correct number of groups, k , by comparing the within-cluster dispersion by that expected under a reference/surrogate set (33). The reference set consisted of 47 artificially formed time series and was created by a random selection of DSA time series segments of 10 time points in length (block bootstrapping). One hundred reference sets are shown to be sufficient in implementations of the gap statistic (34). Within-cluster dispersion calculated for the original DSA dataset and for the mean of the reference data set were compared. For each value of k , a one-standard-error term was calculated. It was used to identify the lowest k for which the difference in within-cluster dispersion between the original and reference data sets stopped increasing. The lowest value of k was subsequently given as the optimal number of clusters.

Stage 4 The remaining shorter time series (*n* = 23) were classified, using the DTW distance measure, into the clusters identified in Stage 3.

### Logistic Regression Analysis

Logistic regression (LR) classification analysis (Matlab, stepwiseglm tool) was performed to consider the associations of identified groups with 5-years graft outcomes whilst accounting for the other potentially confounding variables. Each model was formed via a stepwise LR algorithm with the F-test (35), comparing the fit of two models at each step and determining if variables should be introduced (F-test, *p* < 0.05) into the model or removed (F-test, *p* > 0.1). Due to class imbalance in data, i.e., 75 no-GFs in negative class versus 13 GFs in positive/minority class, two area under curve (AUC) measures were calculated for each model to evaluate LR performance: the receiver operator characteristic (ROC) AUC and precision-recall (PR) AUC. The former is a standard model performance measure but tends to be optimistic on imbalanced classification problems with fewer samples in the minority class. The latter is focused on the minority class and, thus, helps evaluate the model on it. Finally, odds ratios (OR) with corresponding confidence intervals (CI) were evaluated by the t-test (35).

## Results

### Patients Cohort Characteristics

Of the 88 cases, 48% experienced acute rejection (see Supplementary Table S1 for types) within the first 30-days, and 15% experienced GF within 5 years following transplantation (Table 1). The number of cases rises to almost a quarter experiencing GF when looking at the cases with early rejection in isolation. Of the 13 GF cases, eight experienced an acute rejection episode (Table 1). On univariate analysis, only younger age at the time of receiving transplantation was associated with worse 5-year graft outcome, but no significant difference was found across different baseline characteristics, e.g., cross-match types, DSA class or DSA count (Table 1).

**TABLE 1:** Comparison of baseline characteristics for GF and no-GF groups (N = 88).

Graft Failure (GF)	No (75)	Yes (13)	p-value	Odds (95% CI)
Continuous, md(r)				
Age, years	43 (36–49)	33 (23–40)	<**0.01**	
Established renal failure, years	13 (3–18)	9 (1.5–15)	0.24	
Pre-Tx DSA levels, kMFI	5.5 (2.9–9.4)	11.6 (3.6–27.8)	0.07	
Categorical, n (%)				
Gender				
Male	28 (37)	6 (46)		
Female	47 (63)	7 (54)	0.55	0.7 (0.21–2.3)
Living donor				
No	7 (9)	0 (0)		
Yes	68 (91)	13 (100)	0.59	n/a
Previous transplants				
No	29 (39)	3 (23)		
Yes	46 (61)	10 (77)	0.36	2.1 (0.53–8.3)
Early acute rejection				
No	41 (55)	5 (38)		
Yes	33 (45)	8 (62)	0.37	1.9 (0.58–6.4)
Crossmatch status				
CDC (-) FC(-) SAB (+)	18 (24)	5 (38)		
CDC (-) FC(+) SAB (+)	44 (59)	3 (23)	0.10	0.25 (0.05–1.1)
CDC (+) FC(+) SAB (+)	13 (17)	5 (38)	0.72	1.4 (0.33–5.8)
DSA HLA class type				
Class I	27 (36)	4 (31)		
Class II	15 (20)	4 (31)	0.46	1.8 (0.39–8.3)
Class I and II	33 (44)	5 (38)	1.00	1 (0.25–4.2)
DSA count				
≤3	45 (60)	8 (62)		
≥4	30 (40)	5 (38)	1.00	0.94 (0.28–3.1)
HLA (A,B,DR) mismatches				
≤3	54 (72)	10 (77)		
≥4	21 (28)	3 (23)	1.00	0.77 (0.19–3.1)
Pre-treatment DFPP				
No	18 (24)	2 (15)		
Yes	57 (76)	11 (85)	0.72	1.7 (0.35–8.6)
Post-transplant DFPP				
No	66 (88)	9 (69)		
Yes	9 (12)	4 (31)	0.10	3.3 (0.83–13)
Post-transplant lymphodepletion				
No	53 (71)	7 (54)		
Yes	22 (29)	6 (46)	0.33	2.1 (0.62–6.8)

N (%) = number of cases (% of cases); md(r) = median (interquartile range). Continuous and ordinal data, e.g., patient’s age at transplantation, treatment duration, etc., were compared using Wilcoxon rank-sum test. The Fisher two-tailed exact test was applied to binary data, e.g., gender, previous transplant (yes/no), etc. Significant variables (*p* < 0.05) in univariate analysis are displayed in bold.

### Post-Transplant DSA Response Types and Patients Characteristics Within the Identified Groups

**FIGURE 3:**
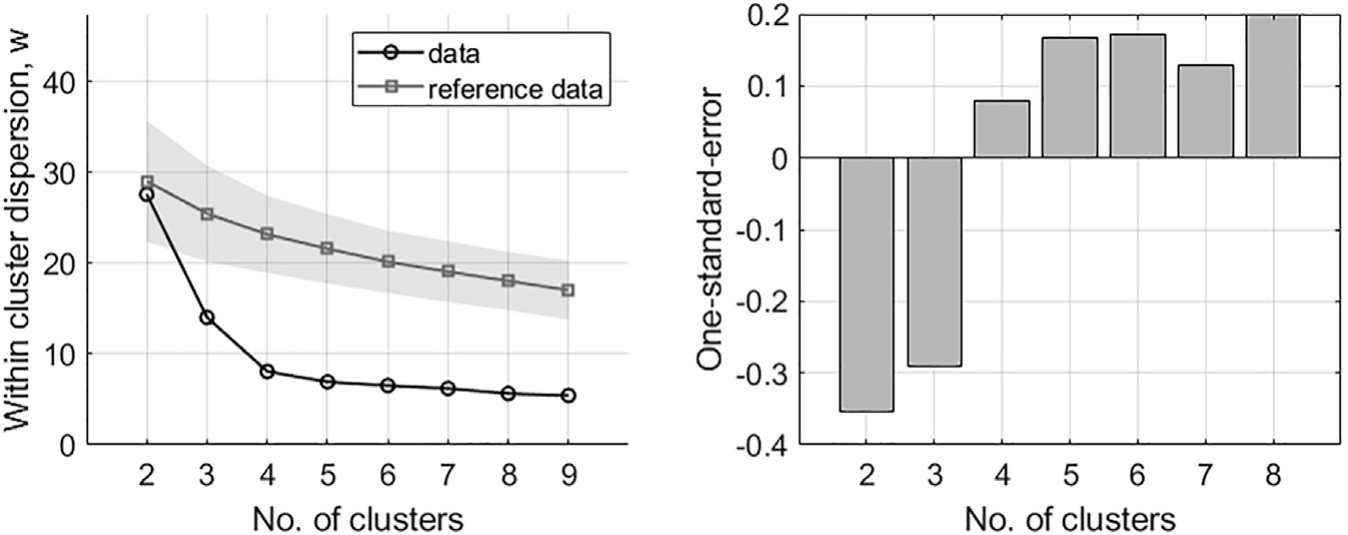
Results of the gap analysis for DSA time series. Left: within-cluster dispersion, the standard deviation is indicated for reference data via the shaded region. Right: one-standard-error measurements. The optimal number of clusters is indicated by the lowest *k* on the left in combination with positive one-standard-error measurement on the right; in this case, *k* = 4.

**FIGURE 4:**
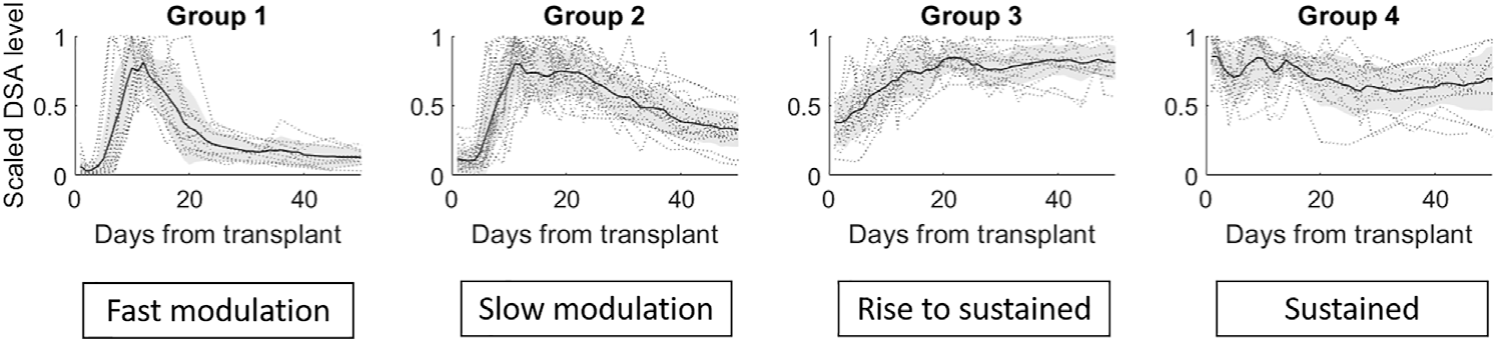
The four groups identified via the gap statistic from DTW agglomerative hierarchical clustering on the DSA dataset. Cluster linkages, i.e., means of the clusters, are shown in dark grey lines. The shaded region displays the standard deviation for each cluster, and individual profiles are displayed in grey dotted lines.

The DTW agglomerative hierarchical clustering of 47 tDSA time series with 50-time points following transplantation are presented in Figure 3. Figure 3 (left figure) shows that at k = 4, the difference within-cluster dispersion between the original and reference data stops increasing, suggesting that the optimal number of clusters is four. This assessment is reinforced when observing the subsequent one-standard error measurement (Figure 3, rightmost figure), whose first positive instance is also at k = 4. A breakdown of the four clusters/groups for post-transplant tDSAs is shown in Figure 4. A description of each group is given, including the additional group of non-responders identified separately in Stage 1 of clustering analysis:

a) No-response group (group 0): low post-transplantation tDSA levels <1500 MFI.
b) Fast modulation (group 1): sharp rise followed by a sharp decrease is tDSA MFI values; the mean peak is day 13 post-transplant. The dynamic behaviour can be summarised as having a short peak duration, typically 3–4 days, before experiencing a sharp drop and settling at the pre-peak DSA level. DSA are typically inactive for up to 5 days following transplant.
c) Slow modulation (group 2): sharp rise followed by a gradual decline in tDSA values; the mean peak day is 13 post-transplant. In this cohort, peak duration is not easily defined and gradually reduces to approximately 30% of peak levels by the 50th-day post-transplant. DSA is typically inactive for up to 5 days following transplant. There are large oscillations in DSA values, not seen in group 1.
d) Rise to sustained (group 3): slower rise followed by sustained high levels; mean peak at day 21 post-transplant, and DSA levels remain consistently high from this point onwards. DSA are typically at a higher baseline than in groups 1 and 2 up to 5 days post-transplant.
e) Sustained (group 4): no substantial rise or fall but tDSA is persistently above 1500 MFI.

**FIGURE 5:**
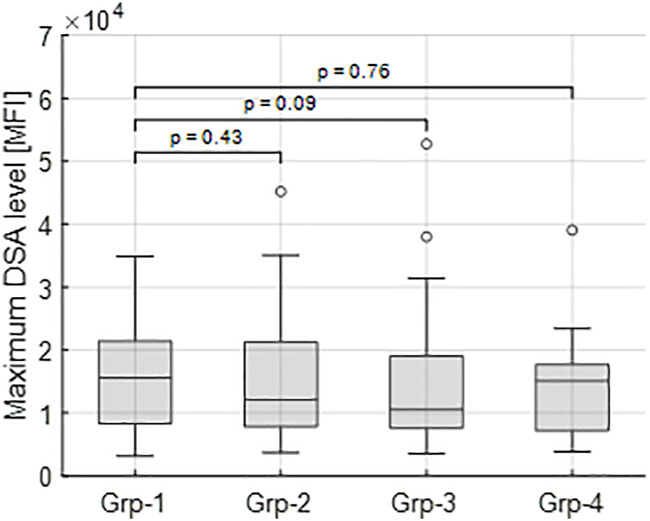
Maximum tDSA level distributions for each group. Group 1 has the highest median value (16,263 MFI) and is compared to groups 2–4 for a significant difference. In each case, no significant differences are identified between groups highlighting how different dynamics occur across all observed DSA ranges.

Inclusion of the remaining 23 shorter DSA time series (Stage 4), classified into the closest of the selected groups 1–4 gave the total numbers of dynamic patterns: group 0 (*n* = 18), group 1 (*n* = 15), group 2 (*n* = 23), group 3 (*n* = 16) and group 4 (*n* = 16). The highest median MFI value (16,263) is observed in group 1; however, there is no significant difference in the maximal DSA MFI values between this group and groups 2–4 (Figure 5).

The patient characteristic analysis of these five groups is illustrated in Table 2. There were no statistically significant differences in the occurrence of pre or post-transplant DFPP (Fisher exact test). Groups were assessed for their associated rates of acute rejection and graft failure. The fast modulation responses (group 1) demonstrated the highest rejection rate of 80%, the sustained group showed the lowest rate at 19%, whereas the slow modulation and rise to sustained groups had rejection rates around 56%. This is in contrast to 5-year GF rates, with 4–7% in the two modulation groups (groups 1 and 2) and 25–31% in the two sustained groups (groups 3 and 4).

**TABLE 2:** Characteristics of groups based on clustering.

	Cluster/group					
	0 No response (*n* = 18)	1 Fast modulation (*n* = 15)	2 Slow modulation (*n* = 23)	3 Rise to sustained (*n* = 16)	4 Sustained (*n* = 16)	All (*n* = 88)
Age (years, mean)	43.94	44.87	40.17	37.13	39.69	41.10
ESRF (years, mean)	13.08	7.33	10.65	12.16	14.23	11.48
Female (%)	55.56	93.33	78.26	31.25	43.75	61.36
LDKT (%)	94.44	100	95.65	93.75	75	92.05
Previous tx (%)	72.22	26.67	60.87	81.25	75	63.64
Crossmatch+ (%)	55.56	73.33	69.57	93.75	81.25	73.86
CDC+ (%)	11.11	13.33	17.39	37.5	25	20.45
Out of all CDC+, CDC>1:2 (%)	0	0	0	100	50	55.56
Pre Tx-DFPP(%)	66.7	80.0	82.6	93.7	62.5	77.27
Average tDSA value	2062	7023	7105	14813	12674	8735
DSA (n)	2	3	3	3	3	3
Rejection within first 30-days, %	16.67	80.0	56.52	56.25	18.75	47.73
Post Tx-DFPP (%)	5.56	33.33	13.04	18.75	6.25	14.77
Lymphocyte-depleting agent (%)	5.56	40	47.83	50	12.5	31.82
GF-5 years (%)	11.11	6.67	4.35	31.25	25	14.77

ESRF, end stage renal failure; LDKT, living donor kidney transplant; Tx, transplant; DFPP, double filtration plasmapheresis; CDC, Complement dependent cytoxicity.

### Modulated Versus Sustained DSA Responses

**FIGURE 6:**
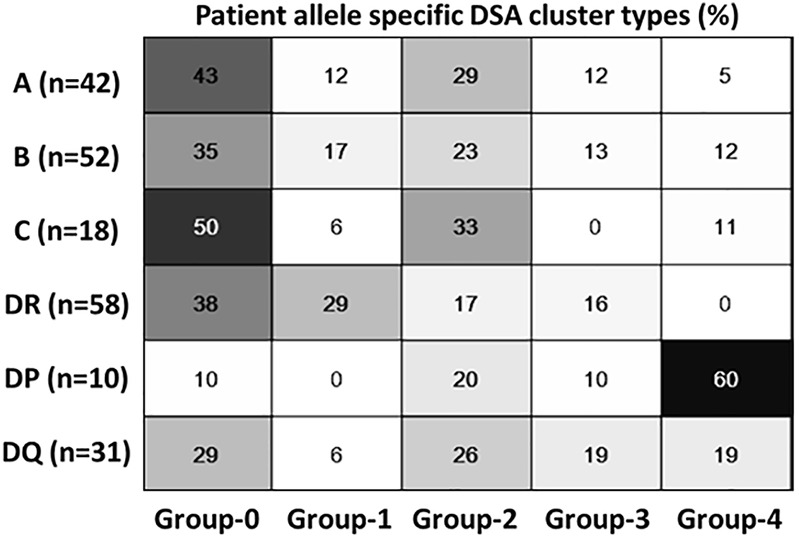
Dynamic cluster patterns at DSA-HLA specific allele levels. Class 1 and DR specific DSA has predominantly modulating response compared to HLA DP; HLA DQ has mixed dynamics.

Because of their similarity in GF rates, the two modulation groups were combined, and the two sustained groups were combined for further analysis. Table 3 illustrates the results of a univariate analysis with significant variables (*p* < 0.05) highlighted in bold. Younger patients and those with previous transplants were more likely to produce a sustained response. In contrast, female gender, more than 4 HLA mismatches and an episode of early acute rejections were associated with a modulated response. The higher pre-treatment tDSA levels were strongly associated with sustained dynamics. Multivariable LR analysis (Table 4), accounting for ten confounding variables with *p* < 0.2 in univariate analysis (Table 3), showed higher pre-treatment tDSA levels, male gender, and the absence of an episode of early acute rejection were all strongly associative of a sustained response (ROC-AUC/baseline = 1.59, PR-AUC/baseline = 1.83). A sub-group analysis of cases who experienced early rejection found no statistically significant difference in treatment proportions between modulated and sustained DSA dynamic responses (p = ns Fischer 2-tail test). The dynamic response varied with DSA specificities with more modulation response for HLA-A, -B and -DR specificities and sustained against HLA-DP specificities (Figure 6), though given patient there were DSA specificities that followed mixed dynamics.

**TABLE 3:** Univariate analysis: comparison of patients’ characteristics for modulation and sustained groups (*n* = 70).

	tDSA dynamic group		p-value	OR (95% CI)
	Modulation (38)	Sustained (32)		
Continuous, md (r)				
Age (years)	44 (36–49)	40 (33–43)	**0.04**	
ESRF (years)	7 (2–16)	15 (7–18)	0.06	
Pre-treatment tDSA level	5200 (3300–9400)	9800 (6600–16000)	**<0.01**	
Maximum tDSA level	12000 (7800–21000)	13000 (7000–18000)	0.87	
Categorical, n (% in each group)				
Gender				
Male	6 (16)	20 (63)		
Female	32 (84)	12 (37)	**<0.001**	0.11 (0.04–0.35)
Living donor				
No	1 (3)	5 (16)		
Yes	37 (97)	27 (84)	0.09	0.15 (0.02–1.3)
Previous transplants				
No	20 (53)	7 (22)		
Yes	18 (47)	25 (78)	**0.01**	4 (1.4–11)
CDC crossmatch				
CDC(-) FC(-) SAB(+)	11 (29)	4 (13)		
CDC(-) FC(+) SAB(+)	21 (55)	18 (56)	0.23	2.4 (0.64–8.7)
CDC(+) FC(+) SAB(+)	6 (16)	10 (31)	0.07	4.6 (0.99–21)
DSA HLA class				
Class I	12 (32)	10 (31)		
Class II	6 (16)	7 (22)	0.73	1.4 (0.35–5.5)
Class I and II	20 (53)	15 (47)	1.00	0.9 (0.31–2.6)
DSA count				
≤3	21 (55)	17 (53)		
≥4	17 (45)	15 (47)	1.00	1.1 (0.42–2.8)
HLA (A, B and DR) mismatches				
≤3	22 (58)	28 (87)		
≥4	16 (42)	4 (13)	**<0.01**	0.2 (0.06–0.67)
Pre-treatment DFPP				
No	7 (18)	7 (22)		
Yes	31 (81)	25 (78)	0.77	0.81 (0.25–2.6)
Early acute rejection				
No	11 (29)	20 (63)		
Yes	27 (71)	12 (37)	**<0.01**	0.24 (0.09–0.67)
5 years graft failure				
No	36 (94)	23 (72)		
Yes	2 (6)	9 (28)	0.02	7 (1.4–36)
Post-transplant DFPP				
No	30 (79)	28 (87)		
Yes	8 (21)	4 (13)	0.53	0.54 (0.15–2)
Post-transplant lymphodepletion				
No	21 (55)	22 (69)		
Yes	17 (45)	10 (31)	0.33	0.56 (0.21–1.5)

n (%) = number of cases (% of cases); md(r) = median (interquartile range). Variables that demonstrated significantly different distributions (*p* < 0.05) within the 2 groups are highlighted in bold.

**TABLE 4:** Multivariable LR model showing association of selected (*p* < 0.2) variables from Table 3 with a sustained dynamic response.

	OR	95% CI	p-value	
Intercept	1.36	2.85	5.97	0.24
Pre-treatment DSA level (↑1000 MFI)	1.08	1.13	1.19	**<0.01**
Gender (Female)	0.06	0.12	0.24	**<0.01**
Acute rejection (Yes)	0.07	0.14	0.28	**<0.01**
Cases	70			
of which are Sustained	32 (46%)			
PR-AUC (PR-AUC/baseline)	0.84 (1.83)			
ROC-AUC (ROC-AUC/baseline)	0.86 (1.59)			

Significant p-values (*p* < 0.05) are highlighted in bold.

**FIGURE 7:**
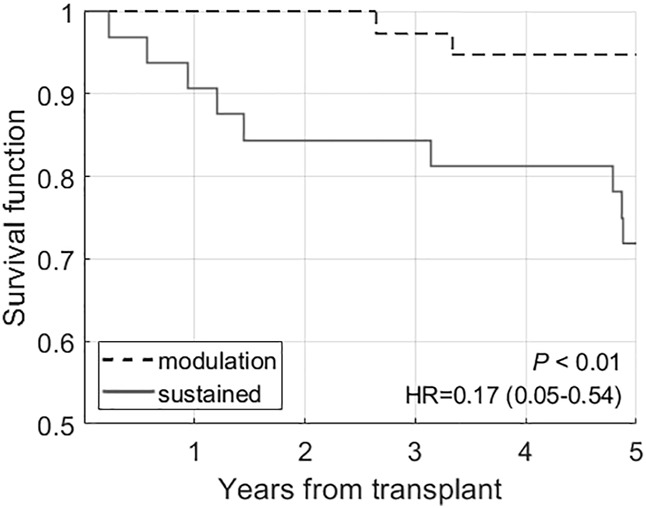
Kaplan-Meier analysis comparing the survival rates in modulation (groups 1 and 2) and sustained (groups 3 and 4) groups.

Kaplan-Meier analysis (Figure 7) confirms that the sustained group has a worse 5-year GF rate. A multivariable logistic regression model was also developed (Table 5) to look at the association of the dynamic patterns of the identified groups, combined with confounding variables, namely age, cross-match status and post-transplant DFPP (from Table 3, *p* < 0.2), with GF. The model in Table 5 shows significant associations of younger age, sustained tDSA response and post-transplant DFPP, with GF (PR-AUC/baseline = 3.83).

**TABLE 5:** Multivariable LR model showing association of DSA response type (modulation and sustained) with 5-year GF while accounting for confounding variables, *p* < 0.2, identified from univariate analysis in Table 3.

	OR	95% CI	p-value	
Intercept	8.29	1.32	52.20	0.25
Age (↑)	0.90	0.86	0.95	**0.03**
DSA response (sustained)	10.05	3.75	26.92	**0.02**
Post-transplant DFPP (no)	0.15	0.05	0.39	**0.05**
Cases	70			
of which are GF	11 (16%)			
PR-AUC (PR-AUC/baseline)	0.60 (3.83)			
ROC-AUC (ROC-AUC/baseline)	0.85 (1.00)			

Significant p-values (*p* < 0.05) are highlighted in bold.

## Discussion

Following kidney transplantation, DSA monitoring can be a useful surrogate marker as allorecognition and memory responses to the re-exposure of HLA is associated with DSA rise and rejection (36). The response can be variable (37) and may depend on the type of sensitisation events (38), age, baseline immunosuppressant, time since sensitisation and level of cross-match before transplantation. T-cell help (39) is required to reactivate memory B-cells (40, 41), and successful treatment of rejection with OKT3/ATG in the study supports this. Equally, a rising DSA trend is often considered more worrying and clinically useful than a steady state or drop in the MFI values.

As a result, most transplant centres and national guidelines suggest post-transplant DSA monitoring (42). However, recommendations vary, and there is much uncertainty over how to perform such monitoring. We have previously described dynamic patterns seen in individual DSA and third party HLA antibodies using the visual description from the same dataset (25), including more complex mathematical modelling of a modulatory type of dynamic behaviour (26). In this study, we have, for the first time, applied an unsupervised clustering (33) approach to the DSA MFI time series to describe the overall dynamic trends and patterns of DSA following HLA-incompatible kidney transplantation and their associations with transplant outcomes, in particular with 5-year graft survival. Four dynamic patterns were identified after separating the non-responder group, demonstrating heterogeneous dynamic behaviour. The total DSA levels remained notably subdued up to the first five to 10 days following transplantation, particularly in fast (group 1) and slow (group 2) modulation groups. This effect in the first 4 days following transplant has been noted in several studies before (18, 25, 43), in part caused by adsorption of HLA antibodies onto the kidney allograft (25). As the use of post-transplant DFPP was not associated with a particular dynamic pattern/group (Tables 2, 3), it suggests that other mechanisms are responsible for differential behaviour.

Our data show that the more sensitised cases at baseline, i.e., those with the higher DSA MFI levels and CDC titre>1:2, are likely to develop the sustained post-transplant response. These, in turn, have the poorest five-year survival. We have previously reported poor survival in the higher titre baseline cases (23). Others have also shown that pre-transplant higher total DSA associates with persistent high total DSA post-transplantation (44) and that sustained total DSA levels associate with the worse outcome than resolved DSA (44, 45, 46). Unlike previous study (45), a single point day 30 DSA levels (tDSA >2000 MFI) in a multivariable model was not an independently significant predictor for graft outcome in our study. We found that early modulation cases are likely to have a very different outcome, despite a significantly higher incidence of early AMR. This all points to different levels of immune regulation in the two broad antibody dynamic groups that we have identified. The very rapid modulation seems in part to be AMR-dependent (87.7% incidence in the fast modulators, Group 1) which implies an active immunological process. Notably, the early antibody dynamics were not significantly associated with the use of post-transplant antibody removal. In some cases, we observed no sustained fall in MFI values over a course of DFPP, and in others, we saw spontaneously MFI falls without DFPP use. A recent study (22) also showed that declining DSA levels following AMR associated with the good longer-term outcome, but the two cohorts are not directly comparable and with different approaches to AMR treatment, so it is unclear whether it is the treatment itself as opposed to the AMR process (and its successful resolution) that determines the outcome.

This study allows some observations to be made that might assist further investigations. First, the “decisions” made by the immune system whether or not to increase DSA levels and then whether or not to have a sustained response or a fall in DSA levels seem to be made in the first 2 weeks or so after transplantation. We did not see late shifts in trajectory, though we cannot exclude the possible impacts of events such as non-adherence and pregnancy. Second, the sustained falls in DSA levels followed initial rises. There was no “sustained to fall” group. This could mean that initial activation of the immune system was required before there could be elimination or suppression of antibody production. Lastly, the rate of fall in DSA levels in our slow modulation group is broadly in line with the known half-life of IgG1 (about 23 days) (47), while the disappearance of DSA in the fast modulation group (half-life in the order of 5 days) seems to imply an active mechanism of DSA removal. However, while the fast disappearance of DSA may be useful, it was not by itself a requirement for good long term outcomes rather the “decision” to modulate was paramount. These observations are speculative but provide many opportunities to drive targeted investigations in the future.

The results of our study reflect specific immunosuppressive protocol, management and monitoring protocols, and case selections, limiting generalizability to broader patient groups. Ideally, a larger multi-centre study is required to confirm the findings. SAB are the best available option for determining DSA levels and have improved our ability to identify and manage allosensitised transplant patients (48, 49). They provide a semi-quantitative measurement of DSA in the form of MFI, with has limitations at larger DSA levels such as the prozone and saturation effects (49–51). While we have made the best efforts to identify and address the prozone and saturation effect occurrences within the cohort, it is still recognised that MFI levels cannot accurately represent true antibody strength. Other limitations include cases discharged back to parent units, a management protocol that may have influenced long term outcomes, and we did not employ protocol biopsies, so we cannot comment on the possible relationship of these to sustained antibody levels. Despite these limitations we made an in-depth and detailed description of DSA dynamic responses. This work may help in future tailoring of treatment so that lower risk HLA-incompatible patients are not subjected to over-immunosuppression even if they have had early acute rejection and that high-risk patients can be looked at more carefully even if they haven’t had an early acute rejection.

## Data Availability

The datasets presented in this article are not readily available because they are available on request and for collaborative research only. Requests to access the datasets should be directed to Natalia Khovanova n.khovanova@warwick.ac.uk.

## Supplementary Material

Supplementary Table S1 Breakdown of acute rejection types (ACR, acute cellular rejection; AMR, antibody mediated rejection; MAR, mixed acute rejection), number of cases (No) and corresponding treatment. [*lymphodepeleting agent–11–OKT3 and 17–ATG (including one with Alemtuzumab)].

## Abbreviations

ACR, acute cellular rejection; AMR, antibody mediated rejection; AUC, area under curve; CDC, complement-dependent cytotoxic; DSA, donor specific antibodies; DFPP, double filtration plasmapheresis; DTW, dynamic time warping; ESRF, end stage renal failure; FC, flow cytometry; GF, graft failure; HLA, human leukocyte antigen; LR, logistic regression; MFI, mean fluorescence intensity; MAR, mixed acute rejection; PR, precision-recall; ROC, receiver operator characteristic; RRT, renal replacement therapy; SAB, single antigen bead; tDSA, total DSA.
