# Supplementary material for: Dynamic Behaviour of Donor Specific Antibodies in the Early Period Following HLA Incompatible Kidney Transplantation
Source: Transpl Int. 2022 Apr 11;35:10128. doi: 10.3389/ti.2022.10128 (PMC9062976; doi:10.3389/ti.2022.10128)
Supplement: Supplementary file 1 [file Table1.docx]

**Supplementary Table**

Table S1**.** Breakdown of acute rejection types (ACR=acute cellular rejection, AMR=antibody mediated rejection, MAR=mixed acute rejection), number of cases (No) and corresponding treatment. (*lymphodepeleting agent - 11 - OKT3 and 17 - ATG (including one with Alemtuzumab))
